# Regional variation in maternal RSV vaccine access and attitudes across two California cohorts

**DOI:** 10.1016/j.pmedr.2026.103408

**Published:** 2026-02-11

**Authors:** Ashley A. Cirillo, Molly Zeme, Amayrani Morales, Neela Rahseparian, Cynthia Cortez, Stephanie L. Gaw, Christine A. Blauvelt

**Affiliations:** a Department of Obstetrics and Gynecology, UCSF Fresno, Fresno, CA, United States; b Division of Maternal Fetal Medicine, Department of Obstetrics, Gynecology & Reproductive Sciences, University of California San Francisco, San Francisco, CA, United States

**Keywords:** Respiratory syncytial virus (RSV), Vaccine, Vaccine uptake, Health disparities, Vaccine hesitancy, Provider communication

## Abstract

**Objective:**

To compare maternal RSV vaccination rates, attitudes, and vaccine information sources between an urban academic hospital in San Francisco and a regional hospital serving a predominantly rural population in Fresno, California.

**Methods:**

Individuals eligible for RSV vaccination during pregnancy were recruited on postpartum units between 11/2024–3/2025 using convenience sampling. Participants completed a survey assessing vaccination status, attitudes, and information sources.

**Results:**

Of 149 approached patients, 94 completed the survey (63.09% response rate; San Francisco, 62/114 [54.39%]; Fresno, 32/35 [91.43%]). San Francisco participants were older (mean [SD], 36.11 [4.06] vs. 29.19 [5.60] years; *p* < 0.01), more often privately insured (87.10% vs. 6.25%; *p* < 0.01), and less likely Hispanic-identifying (6.45% vs. 75.00%; p < 0.01). RSV vaccine uptake was higher in San Francisco than in Fresno (77.42% vs 31.25%; *p* < 0.01). San Francisco participants more often reported receiving vaccine information from healthcare providers (93.55% vs 62.50%; *p* < 0.01). Among unvaccinated participants, lack of awareness (36.36%) and not being offered the vaccine (27.27%) predominated in Fresno, whereas safety concerns (42.86%) predominated in San Francisco.

**Conclusions:**

Marked regional differences in maternal RSV vaccination highlight the need for system-level strategies to ensure equitable vaccine access and education, alongside continued vaccine safety research to build patient confidence.

## Highlights

• Survey of prenatal respiratory syncytial virus (RSV) vaccination at two hospitals.
• Healthcare provider vaccine counseling was more prevalent at the urban site.
• Primary reason for non-vaccination at rural site: Unaware of vaccine or not offered.
• Primary reason for non-vaccination at urban site: Vaccine safety concerns.
• Findings support need for standardized, equitable prenatal vaccine delivery systems.

## 1 Introduction

Respiratory syncytial virus (RSV) infection is the most common cause of infant hospitalization, accounting for 58,000–80,000 hospitalizations and 100–300 deaths annually in the United States (U.S.) and 3.6 million hospitalizations and 100,000 deaths globally among children under 5 years old (Fleming-Dutra et al., 2023; Respiratory syncytial virus (RSV), 2026). In August 2023, the U.S. Food and Drug Administration approved the first maternal RSV vaccine (Abrysvo/Pfizer) to prevent RSV-related morbidity in infants through transplacental antibody transfer, with the Advisory Committee on Immunization Practices recommending seasonal administration between 32 and 36 weeks' gestation (Fleming-Dutra et al., 2023). RSV vaccination in pregnancy has since been endorsed by the Center for Disease Control and Prevention (CDC) (Fleming-Dutra et al., 2023), the American College of Obstetrics and Gynecology (ACOG) (Maternal Respiratory Syncytial Virus Vaccination, 2025), the Society for Maternal-Fetal Medicine (SMFM) (Joseph et al., 2024), and the American Academy of Family Physicians (AAFP) (Obstetric Care Professionals Recommend RSV Vaccine for Pregnant Individuals, 2025).

Early implementation data suggest that RSV vaccine uptake during pregnancy has been low to moderate, with substantial geographic variation. In population-based surveillance studies across 33 U.S. states and the District of Columbia, only 10.4% of eligible pregnant individuals received the RSV vaccine during the 2023–2024 season, with state-level coverage ranging from 1.0% to 21.8% (Boundy et al., 2025). Higher uptake has been reported in selected urban academic centers, with estimates up to 64% (Blauvelt et al., 2025; Son et al., 2024; Litman et al., 2025; DeSilva et al., 2025). National survey data suggest that vaccination intent exceeds actual uptake, underscoring missed opportunities in clinical care and barriers to access (Saper et al., 2024).

The extent to which regional differences in RSV vaccine uptake reflect system-level factors – such as access to vaccines in clinics and standardized education protocols – versus patient-level hesitancy remains unclear. Few studies have examined reasons for maternal RSV vaccine uptake or declination, particularly across different health care systems. Understanding how system-level and patient-level factors interact is essential for designing effective interventions.

We conducted a cross-sectional survey study comparing postpartum patients from two distinct California populations: patients delivering at an urban academic hospital in San Francisco and those delivering at a regional referral center in Fresno. We examined maternal RSV vaccination rates, participants' attitudes toward RSV vaccination in pregnancy, and reported barriers to vaccination.

## 2 Materials and methods

### 2.1 Study design and population

This was a cross-sectional survey study of postpartum patients who were candidates to receive the maternal RSV vaccine during the 2024–2025 season. The study was approved by the Institutional Review Boards at the University of California, San Francisco (UCSF) and Community Regional Medical Center (CRMC), Fresno. Eligible participants were those who had been 32 to 36 weeks' gestation between September 1, 2024 and January 31, 2025. This timeframe was selected because it represented the period during which the RSV vaccine was administered to pregnant individuals. Additional eligibility criteria included English proficiency and delivery at UCSF (San Francisco) or CRMC (Fresno). Participants were recruited using convenience sampling; eligible participants were approached on the postpartum units of both hospitals between November 1, 2024 and March 31, 2025, and invited to complete the survey.

### 2.2 Measures

Written informed consent was obtained and a questionnaire was administered – online or on paper, per participant preference. The survey instrument (**Appendix A**) was developed by the study team to assess self-reported RSV vaccination status, attitudes toward vaccination, and sources of vaccine information. The questionnaire was not a previously validated instrument. Prior to study launch, the survey instrument was pilot-tested with postpartum patients to evaluate question clarity and comprehension. Medical records were reviewed for maternal demographic information, clinical characteristics, and confirmation of vaccine receipt through the California Immunization Registry. Race and ethnicity were analyzed as separate, mutually exclusive variables.

Maternal clinical characteristics obtained from medical records included: age, parity, chronic hypertension (elevated blood pressure diagnosed prior to pregnancy or before 20 weeks' gestation), cardiovascular disease (e.g. history of structural heart disease, cardiomyopathy, or other chronic cardiovascular condition), asthma, pulmonary disease (e.g. chronic obstructive pulmonary disease, interstitial lung disease, or other chronic pulmonary condition), pregestational diabetes mellitus (diabetes diagnosed prior to pregnancy or hemoglobin A1C ≥6.5% prior to 14 weeks' gestation), gestational diabetes (abnormal glucose tolerance testing after 14 weeks' gestation), pregestational body mass index (BMI) (patient-reported BMI prior to pregnancy or earliest documented BMI during pregnancy), assisted reproductive technologies (e.g. in vitro fertilization), and multiple gestation in the current pregnancy.

### 2.3 Statistical analysis

Differences between sites were compared using Fisher exac*t-*tests for categorical variables and *t-*tests for continuous variables. Missing data were minimal and limited to a single missing value for maternal age; all analyses used available data with pairwise deletion, and no imputation was performed. Statistical significance was defined as 2-sided *p*-value <0.05. Analyses were conducted using R version 4.5.1 (R Foundation).

## 3 Results

Of 149 eligible participants who were approached, 94 completed the survey (63.09% overall response rate): 62 of 114 (54.39%) in San Francisco and 32 of 35 (91.43%) in Fresno. Demographic characteristics of the respondents are summarized in Table 1. San Franscico participants were older (mean [SD] age, 36.11 [4.06] vs. 29.19 [5.60] years; *p* < 0.01), were more likely to have private insurance (87.10% vs 6.25%; p < 0.01), and most often identified as White (56.45%), Asian (32.26%), and non-Hispanic (93.55%). In contrast, participants in Fresno most often identified as White (84.38%) and Hispanic (75.00%). Participants in San Francisco were more often nulliparous (56.45% vs 31.25%; *p* = 0.03) and used assisted reproductive technologies (22.58% vs 0.00%; *p* < 0.01). Most maternal medical comorbidities were similar between sites, with the exception of obesity, which was more common among Fresno participants (59.38% vs 27.42%; *p* < 0.01).

**Table 1:** Patient characteristics. Differences between sites were compared using Fisher's exac*t-*tests for categorical variables and *t-*tests for continuous variables.

Variable	All (*n* = 94)	San Francisco (*n* = 62)	Fresno (*n* = 32)	p-value
Age – years, mean (SD)	33.81 (5.65)	36.11 (4.06)	29.19 (5.60)	p < 0.01
Nulliparous	45 (47.87)	35 (56.45)	10 (31.25)	p = 0.03

Insurance status				
Private	56 (59.57)	54 (87.10)	2 (6.25)	p < 0.01
Public	36 (38.30)	7 (11.29)	29 (90.63)	p < 0.01
Unknown	2 (2.13)	1 (1.61)	1 (3.13)	p = 1.00

Race				
African American or Black	6 (6.38)	2 (3.23)	4 (12.50)	*p* = 0.18
American Indian or Alaska Native	1 (1.06)	1 (1.61)	0 (0.00)	p = 1.00
Asian	20 (21.28)	20 (32.26)	0 (0.00)	p < 0.01
Native Hawaiian or Pacific Islander	1 (1.06)	1 (1.61)	0 (0.00)	*p* = 1.00
White	62 (65.96)	35 (56.45)	27 (84.38)	p = 0.01
Other	1 (1.06)	0 (0.00)	1 (3.13)	*p* = 0.34
Unknown	3 (3.19)	3 (4.84)	0 (0.00)	*p* = 0.55

Ethnicity				
Hispanic	28 (29.79)	4 (6.45)	24 (75.00)	p < 0.01
Non-Hispanic	66 (70.21)	58 (93.55)	8 (25.00)	

Maternal medical conditions				
Chronic hypertension	8 (8.51)	5 (8.06)	3 (9.38)	p = 1.00
Cardiovascular disease	0 (0.00)	0 (0.00)	0 (0.00)	N/A
Asthma	12 (12.77)	9 (14.52)	3 (9.38)	*p* = 0.75
Pulmonary disease	2 (2.13)	2 (3.23)	0 (0.00)	p = 0.55
Pregestational diabetes mellitus	3 (3.19)	2 (3.23)	1 (3.13)	*p* = 1.00
Gestational diabetes mellitus	6 (6.38)	4 (6.45)	2 (6.25)	p = 1.00
Body Mass Index >30	36 (38.30)	17 (27.42)	19 (59.38)	p < 0.01
Assisted reproductive technologies	14 (14.89)	14 (22.58)	0 (0.00)	p < 0.01
Multiple gestation	1 (1.06)	1 (1.61)	0 (0.00)	p = 1.00

Maternal vaccinations				
COVID-19 vaccine (lifetime)	75 (79.79)	57 (91.94)	18 (56.25)	p < 0.01
COVID-19 booster (prenatal)	40 (42.55)	39 (62.90)	1 (3.13)	*p* < 0.01
Influenza vaccine (prenatal)	56 (59.57)	51 (82.26)	5 (15.63)	*p* < 0.01 p < 0.01
Tdap vaccine (prenatal)	77 (81.91)	58 (93.55)	19 (59.38)	

Newborn medications				
Hepatitis B vaccine	73 (77.66)	57 (91.94)	16 (50.00)	p < 0.01
Erythromycin ointment	89 (94.68)	60 (96.77)	29 (90.63)	*p* = 0.33
Vitamin K injection	90 (95.74)	60 (96.77)	30 (93.75)	*p* = 0.60

**Fig. 1:**
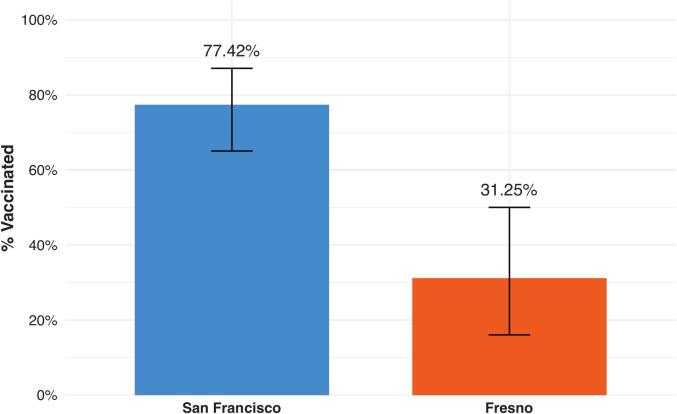
RSV Vaccine Uptake by Site. Bars represent the proportion of participants who received the RSV vaccine during pregnancy among those enrolled at each site. Error bars represent 95% confidence intervals calculated using the Clopper-Pearson exact method.

**Fig. 2:**
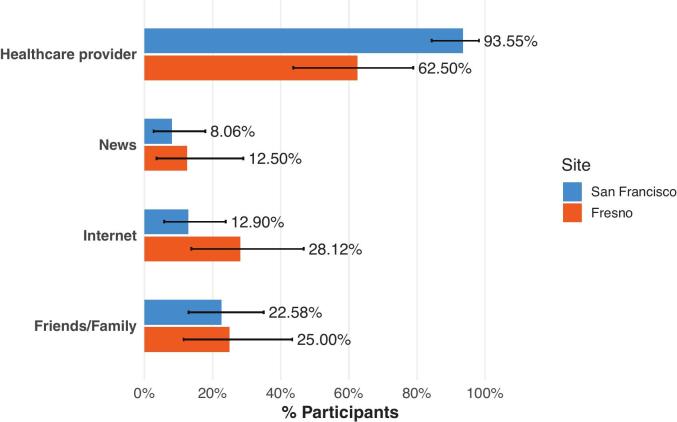
Sources of Vaccine Information by Site. Participants were asked, *“How did you learn about the RSV vaccine during your pregnancy?”* and could select more than one response. Bars represent the percentage of participants at each site who reported each source of information. Error bars indicate 95% confidence intervals calculated using the Clopper–Pearson exact method.

RSV vaccine uptake was significantly higher among participants delivering in San Francisco than those delivering in Fresno (77.42% vs 31.25%; *p* < 0.01) (Table 2 and Fig. 1). There were substantial differences in how vaccine information was obtained (Table 2 and Fig. 2). Participants in San Francisco were significantly more likely to report learning about the RSV vaccine from a healthcare provider than those in Fresno (93.55% vs 62.50%, *p* < 0.01). Other sources of information, including news, internet, and friends or family, did not differ significantly between sites.

**Table 2:** Survey Data. Differences between sites were compared using Fisher's exact tests.

Variable	All (n = 94)	San Francisco (n = 62)	Fresno (n = 32)	p-value
RSVpreF vaccination received	58 (61.70)	48 (77.42)	10 (31.25)	p < 0.01
How did you learn about the RSV vaccine?				
Healthcare provider	78 (82.98)	58 (93.55)	20 (62.50)	p < 0.01
News	9 (9.57)	5 (8.06)	4 (12.50)	*p* = 0.48
Internet	17 (18.09)	8 (12.90)	9 (28.13)	*p* = 0.09
Friends/Family	22 (23.40)	14 (22.58)	8 (25.00)	*p* = 0.80
Other	1 (1.06)	0 (0.00)	1 (3.13)	p = 0.34

Vaccinated				
Primary reason for receiving the vaccine				
Healthcare provider recommendation	16 (27.59)	14 (29.17)	2 (20.00)	*p* = 0.71
Protect pregnant person against RSV	1 (1.72)	1 (2.08)	0 (0.00)	p = 1.00
Protect baby against RSV	39 (67.24)	32 (66.67)	7 (70.00)	*p* = 1.00
Other	2 (3.45)	1 (2.08)	1 (10.00)	*p* = 0.32

Unvaccinated				
Have heard about the prenatal RSV vaccine	28 (77.78)	13 (92.86)	15 (68.18)	p = 0.12
Primary reason for not receiving the vaccine				
Did not know about the vaccine	9 (25.00)	1 (7.14)	8 (36.36)	*p* = 0.06
Vaccine was not offered to me	9 (25.00)	3 (21.43)	6 (27.27)	p = 1.00
Unable to get a vaccine appointment	1 (2.78)	0 (0.00)	1 (4.55)	*p* = 1.00
Wanted infant to get monoclonal Ab instead	0 (0.00)	0 (0.00)	0 (0.00)	N/A
Do not believe RSV is a serious disease	1 (2.78)	0 (0.00)	1 (4.55)	p = 1.00
Concerns about safety of vaccine for baby	7 (19.44)	6 (42.86)	1 (4.55)	p < 0.01
Concerns about safety of vaccine for me	1 (2.78)	0 (0.00)	1 (4.55)	p = 1.00
Worried about vaccine side effects	1 (2.78)	1 (7.14)	0 (0.00)	p = 0.39
Concerns about vaccines in general	2 (5.56)	0 (0.00)	2 (9.09)	*p* = 0.51
Fear of needles	1 (2.78)	1 (7.14)	0 (0.00)	*p* = 0.39
Other	4 (11.11)	2 (14.29)	2 (9.09)	*p* = 0.63

Do you plan to give your baby the RSV monoclonal Ab?				
Yes	13 (36.11)	9 (64.29)	4 (18.18)	p = 0.01
No	13 (36.11)	3 (21.43)	10 (45.45)	p = 0.18
Not sure	10 (27.78)	2 (14.29)	8 (36.36)	*p* = 0.26

**Fig. 3:**
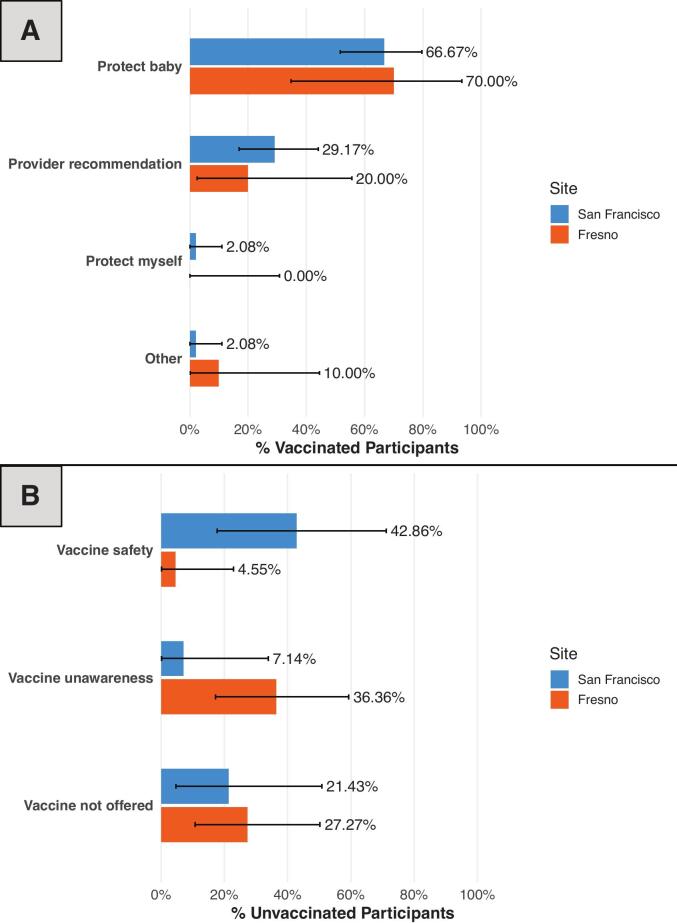
Primary Reasons Reported for Receiving or Not Receiving the Vaccine. Vaccinated participants were asked, *“What was the most important factor in your decision to receive the RSV vaccine?”* and unvaccinated participants were asked, *“What was the most important factor in your decision not to receive it?”* Bars represent the percentage of participants at each site who reported each **A)** Primary reason for receiving the vaccine, and **B)** Primary reason for not receiving the vaccine. Error bars indicate 95% confidence intervals calculated using the Clopper–Pearson exact method.

Among vaccinated participants at both sites, the most frequently cited reason for vaccination was to protect the baby from RSV (67.24%), followed by healthcare provider recommendation (27.59%) (Fig. 3A). Among unvaccinated participants, those in Fresno were more likely to report not having heard about the vaccine than those in San Francisco (31.82% vs 7.14%, *p* = 0.12). The top reported reasons for not receiving a vaccine among Fresno participants were not knowing about the vaccine (36.36%) or not being offered the vaccine (27.27%). In contrast, unvaccinated participants in San Francisco most often cited concerns about vaccine safety for the baby (42.86% vs 4.55%, *p* < 0.01) as the primary reason for nonreceipt. (Fig. 3B).

San Francisco participants also had higher uptake rates of other recommended vaccines (Table 1), including any COVID-19 vaccine including pre-pregnancy (91.94% vs 56.25%; *p* < 0.01), and vaccines given during pregnancy including a COVID-19 booster (62.90% vs 3.13%; *p* < 0.01), influenza (82.26% vs 15.63%; p < 0.01), and tetanus-diphtheria-pertussis (Tdap) (93.55% vs 59.38%; p < 0.01). Neonates of San Francisco participants were also more likely to receive a hepatitis B vaccine prior to discharge (91.94% vs 50.00%; p < 0.01).

Among unvaccinated participants, whose infants were therefore eligible for the RSV monoclonal antibody, 64.29% in San Francisco reported planning to give their baby the monoclonal antibody compared with only 18.18% in Fresno (*p* = 0.01) (Table 2).

## 4 Discussion

In this cross-sectional survey study comparing two distinct California cohorts, we found significant regional differences in maternal RSV vaccine uptake, sources of vaccine information, and attitudes toward vaccination. Despite both sites being academic referral centers, participants delivering in San Francisco had more than double the vaccine uptake than participants delivering in Fresno. Intentions regarding neonatal RSV monoclonal antibody prophylaxis also diverged, with unvaccinated San Francisco participants more likely to plan administration than those in Fresno. Reasons for RSV non-vaccination differed by site: Fresno participants most often cited lack of awareness of the vaccine or not being offered it, whereas San Francisco participants most commonly expressed concerns about vaccine safety.

Our findings align with prior work demonstrating that access, provider recommendation, and perceived safety are central determinants of RSV vaccine uptake. In a U.S. survey conducted during the inaugural 2023–2024 season, only one-third of pregnant individuals reported receiving the RSV vaccine, with nonavailability (40%) and lack of provider recommendation (22%) as the predominant barriers (Shedlock et al., 2025; Zeme and Gaw, 2025). Similarly, a 2024 survey in England and Scotland found that one-third of unvaccinated patients had significant difficulty accessing vaccination sites – highlighting logistical constraints to maternal vaccination (Williams et al., 2025). Multiple studies have underscored that trust in healthcare providers and reassurance regarding safety and efficacy are strong motivators for acceptance of maternal RSV vaccination (Shedlock et al., 2025; Gagnon et al., 2025; Tucker et al., 2024; Callaghan et al., n.d.; Gavaruzzi et al., 2025; Wang et al., 2025). In a U.S. phone survey of individuals who declined maternal RSV vaccination, 80% expressed general distrust of the new vaccine and nearly half cited concerns about fetal harm (Tucker et al., 2024). Notably, 94% of respondents in that study felt they had received adequate counseling from a clinician, suggesting that a provider recommendation may not fully overcome apprehension about new vaccines. Collectively, these findings and ours underscore that both structural barriers (such as access and provider offer) and perceptual barriers (such as safety concerns and distrust of new vaccines) shape maternal RSV immunization behavior.

Wide regional variation has been documented for other vaccinations during pregnancy including influenza, Tdap, and COVID-19 (Kortsmit et al., 2024; Kaur et al., 2023; Zerbo et al., 2020; Rowe et al., 2025). In the 2020 Pregnancy Risk Assessment Monitoring System (PRAMS) dataset, influenza vaccine coverage ranged from 35% in Puerto Rico to 80% in Massachusetts, with higher-coverage states implementing coordinated education campaigns, community partnerships, culturally tailored messaging, and reimbursement programs that incentivized vaccine stocking and administration (Kortsmit et al., 2024). Conversely, lower coverage has been consistently observed among younger, publicly insured, racially minoritized, and rural populations – reflecting disparities in provider offer, mistrust in the healthcare system, and broader structural barriers (Kortsmit et al., 2024; Kaur et al., 2023; Zerbo et al., 2020; Rowe et al., 2025; Daley et al., 2023). Our findings parallel these patterns and illustrate how health system integration and patient characteristics may shape maternal vaccination behaviors. The patient population at UCSF is predominantly privately insured, and the majority receive prenatal care through UCSF-affiliated clinics where the RSV vaccine was stocked and routinely offered at 32–36 weeks' gestation. In contrast, CRMC serves a predominantly lower income population, with over 90% publicly insured. Prenatal care for patients delivering at CRMC is fragmented across more than 20 clinics, including UCSF-affiliated clinics in Fresno, federally qualified health centers, and private practices. To our knowledge, only one Fresno-area prenatal clinic stocked the RSV vaccine during the 2024–2025 season. Thus, most patients seeking RSV vaccination were referred to commercial pharmacies, a barrier known to reduce vaccine uptake. These system-level differences likely contributed to the observed disparity in vaccine knowledge and uptake, with integrated health systems and reliable on-site vaccine availability facilitating uptake in San Francisco, while fragmented care delivery and limited on-site stocking posed barriers in Fresno.

The marked discrepancy in RSV vaccine awareness and offer between our study populations highlights an actionable opportunity for intervention. At the patient-level, a strong and clear recommendation from a trusted clinician – particularly OB-GYNs and midwives – remains the single most effective driver of vaccine uptake (Kennedy et al., 2012; Razai et al., 2024; Razai et al., 2023; *Fertil. Steril.*, 2024). Pregnant people are nearly six times more likely to be vaccinated against influenza when a provider recommendation is given (69% vs 12%) (Kennedy et al., 2012). Tailored face-to-face counseling that emphasizes maternal and fetal protection and directly addresses safety concerns, combined with written or digital reminders, can further mitigate vaccine hesitancy and correct misinformation (Razai et al., 2024; Razai et al., 2023; *Fertil. Steril.*, 2024; Baïssas et al., 2021). Provider-focused interventions – such as vaccine education and communication training – can strengthen clinicians' ability to give consistent vaccine recommendations while addressing patient concerns with confidence and cultural sensitivity. Finally, at the system-level, ensuring vaccine availability on-site during prenatal visits is the most important intervention to increase uptake. Prior studies have shown that pregnant people are more than twice as likely to receive an influenza vaccine if they are directly offered vaccination than those who are not (75% vs 34%) (Kennedy et al., 2012). In our study, unvaccinated individuals in Fresno were far less likely to report discussing or being offered the RSV vaccine, mirroring the absence of routine on-site availability in most Fresno-area prenatal clinics. Use of standing vaccine orders and incorporation of reminders into clinical workflows can further reduce missed opportunities. Together, these multi-level interventions—patient, provider, and system—can meaningfully improve maternal RSV vaccine coverage and reduce regional disparities.

Future studies should use in-depth qualitative methods including semi-structured interviews and focus groups to better characterize the drivers of RSV vaccine uptake in pregnancy. Additionally, implementation science studies could clarify how clinic workflows – such as vaccine stocking practices and ordering pathways – influence how and when patients receive the RSV vaccine. Finally, ongoing accumulation of safety data is likely to provide reassurance to patients concerned about vaccine safety (Blauvelt et al., 2025; Son et al., 2024; Madhi et al., 2025; Phijffer et al., 2024).

This study is among the first to examine attitudes toward the newly approved maternal RSV vaccine. While influenza and Tdap vaccination during pregnancy are well studied, it remains uncertain whether the same attitudes and barriers extend to RSV; our findings provide early insight into this evolving landscape. Importantly, we explored patient-level attitudes in two distinct California cohorts – one with relatively high uptake and one with low uptake – allowing comparison of how health system factors and patient-level factors may influence vaccine uptake. Other strengths of our study include confirmation of vaccine receipt through the California Immunization Registry and integration of survey data with chart-abstracted clinical data.

Several limitations should be acknowledged. Our response rate of 63.09% raises the possibility of nonresponse bias, particularly among San Francisco participants where the response rate was 54.39%. Individuals who elected to complete the survey may differ systematically from those who declined. Responses may also be subject to recall and social desirability bias; for example, vaccinated participants may have been more likely to recall a provider recommendation or offer, potentially overestimating the strength of clinician engagement. Additionally, the survey instrument was investigator-developed and not previously validated; however, the questionnaire was pilot-tested prior to deployment to enhance clarity. Eligibility was restricted to English-speaking patients, limiting generalizability to linguistically diverse populations who may face additional barriers to vaccination. Finally, analyses were unadjusted and residual confounding by demographic and socioeconomic factors is possible. However, high collinearity between site and key sociodemographic characteristics, along with the limited sample size, precluded stable multivariable modeling. Larger, multi-site studies incorporating diverse geographic regions, languages, insurance types, and care models are needed to validate and extend these findings.

## 5 Conclusions

We observed significant differences in maternal RSV vaccine uptake, awareness, and attitudes between two California cohorts. In Fresno, low uptake was largely driven by lack of vaccine awareness and lack of provider offer, whereas in San Francisco, uptake was higher but safety concerns predominated among those who declined vaccination. Our findings underscore the need to proactively address structural barriers, such as ensuring on-site vaccine availability across prenatal clinics and implementing national vaccine education initiatives rather than relying on fragmented, clinic-specific approaches. In parallel, continued research in pregnant populations is critical to provide robust safety data on vaccinations in pregnancy. As maternal RSV vaccination uptake remains low nationally, understanding and addressing barriers across diverse settings is essential to achieving equitable and effective implementation.

## CRediT authorship contribution statement

**Ashley A. Cirillo:** Writing – original draft, Methodology, Data curation, Conceptualization. **Molly Zeme:** Data curation. **Amayrani Morales:** Data curation. **Neela Rahseparian:** Data curation. **Cynthia Cortez:** Project administration, Data curation. **Stephanie L. Gaw:** Writing – review & editing, Supervision, Methodology, Conceptualization. **Christine A. Blauvelt:** Writing – original draft, Methodology, Formal analysis, Conceptualization.

## Ethical approval

This study was approved by the Institutional Review Boards at the University of California, San Francisco (UCSF) and Community Regional Medical Center (CRMC), Fresno (IRB #24-41601, approved 10/3/2024).

## Declaration of competing interest

The authors declare that they have no known competing financial interests or personal relationships that could have appeared to influence the work reported in this paper.

## Data Availability

Data will be made available on request.

## Appendix A Supplementary data

Supplementary material Survey Instrument Supplementary material
