# Supplementary material for: Regional variation in maternal RSV vaccine access and attitudes across two California cohorts
Source: Prev Med Rep. 2026 Feb 11;63:103408. doi: 10.1016/j.pmedr.2026.103408 (PMC12933853; doi:10.1016/j.pmedr.2026.103408)
Supplement: Supplementary material — Survey Instrument [file mmc1.pdf]

RSV Vaccine Study - Follow-up Survey

Thank you for your participation in this study. Please use the link below to complete a brief survey about your experiences with the RSV vaccine for pregnant individuals and the RSV monoclonal antibody for infants. This survey should take less than 10 minutes to complete.

Please email christine.blauvelt@ucsf.edu or stephanie.gaw@ucsf.edu with any questions.

Thank you!

Respiratory Syncytial Virus (RSV) is a virus that can cause infections of the lungs and breathing passages. RSV is a leading cause of hospitalization in infants during their first year of life.

In 2023, two treatments became available to prevent severe RSV disease in infants:
1) A vaccine given to pregnant people between 32-36 weeks' gestation
2) A monoclonal antibody injection given to infants in the first 8 months of life

This survey will ask you questions about the RSV vaccine for pregnant people and the RSV monoclonal antibody injection for infants.

Did you receive the RSV vaccine during your pregnancy?

- ◯ Yes
- ◯ No
- ◯ Not sure

Have you heard about the RSV vaccine for pregnant people?

- ◯ Yes
- ◯ No

How did you learn about the RSV vaccine during your pregnancy? (Check all that apply)

- ☐ Healthcare provider
- ☐ News
- ☐ Internet
- ☐ Friends/Family
- ☐ Other (please specify)

From what other source did you learn about the RSV vaccine?

____

Did you experience any of these symptoms after receiving the RSV vaccine? (Check all that apply)

- ☐ Fever
- ☐ Chills
- ☐ Pain at the injection site
- ☐ Redness at the injection site
- ☐ Rash in the immediate area surrounding the injection point
- ☐ Rash not in the immediate area surrounding the injection point
- ☐ Headache
- ☐ Joint pains
- ☐ Muscle aches or body aches
- ☐ Fatigue or tiredness
- ☐ Nausea
- ☐ Vomiting
- ☐ Diarrhea
- ☐ Abdominal pain
- ☐ Uterine contractions
- ☐ Vaginal bleeding
- ☐ Decreased movement of the baby
- ☐ Any other symptoms or health concerns (please specify)
- ☐ None of the above

What other symptoms did you experience after receiving the RSV vaccine?

____

What was the most important factor in your decision to receive the RSV vaccine? (Select one)	◯ My healthcare provider recommended it.<br>◯ I wanted to protect myself against RSV.<br>◯ I wanted to protect my baby against RSV.<br>◯ Other reason (specified above)
Why did you decide not to receive the RSV vaccine during your pregnancy? (Check all that apply)	☐ I did not know about the vaccine.<br>☐ The vaccine was not offered to me.<br>☐ I was unable to get a vaccine appointment.<br>☐ I wanted my infant to get the RSV monoclonal antibody instead<br>☐ I do not believe RSV is a serious disease.<br>☐ I had concerns about safety of the vaccine for my baby.<br>☐ I had concerns about safety of the vaccine for me.<br>☐ I worried about vaccine side effects.<br>☐ I have concerns about vaccines in general.<br>☐ I have a fear of needles.<br>☐ Other (please specify)
What were the other reasons you decided not to get the RSV vaccine?	__________________________________
What was the most important factor in your decision not to receive the RSV vaccine during your pregnancy? (Select one)	◯ I did not know about the vaccine.<br>◯ The vaccine was not offered to me.<br>◯ I was unable to get a vaccine appointment.<br>◯ I wanted my infant to get the RSV monoclonal antibody instead<br>◯ I do not believe RSV is a serious disease.<br>◯ I had concerns about safety of the vaccine for my baby.<br>◯ I had concerns about safety of the vaccine for me.<br>◯ I worried about vaccine side effects.<br>◯ I have concerns about vaccines in general.<br>◯ I have a fear of needles.<br>◯ Other reason (specified above)
Did or do you plan to have your infant receive the RSV monoclonal antibody injection (nirsevimab/Beyfortus)?	◯ Yes<br>◯ No<br>◯ Not sure
Why did you decide to give your baby the RSV monoclonal antibody? (Check all that apply)	☐ My healthcare provider recommended it<br>☐ It was recommended by public health guidelines (CDC and AAP)<br>☐ I wanted to protect my baby against RSV<br>☐ Other reason (please specify)
What were the other reasons you decided to give your baby the RSV monoclonal antibody?	__________________________________
Why did you decide not to give your baby the RSV monoclonal antibody? (Check all that apply)	☐ My baby was not offered the monoclonal antibody.<br>☐ I had concerns about the safety of the monoclonal antibody for my baby.<br>☐ I worried about medication side effects.<br>☐ I do not believe RSV is a serious disease.<br>☐ Other (please specify)
What were the other reasons you decided not to give your baby the RSV monoclonal antibody?	__________________________________

Is there anything else you'd like us to know about your experience with the RSV vaccine for pregnant individuals or the RSV monoclonal antibody for infants? ____
